# Combined approach to treatment of advanced stages of medication-related osteonecrosis of the jaw patients

**DOI:** 10.1016/j.bjorl.2021.04.004

**Published:** 2021-05-06

**Authors:** Onur Şahin, Ender Akan, Birkan Tatar, Ceren Ekmekcioğlu, Nuri Ünal, Onur Odabaşı

**Affiliations:** a İzmir Katip Çelebi University, Faculty of Dentistry, Department of Oral and Maxillofacial Surgery, İzmir, Turkey; b Izmir Katip Celebi University, Faculty of Dentistry, Department of Prosthodontics, Izmir, Turkey; c Private Practice, İzmir, Turkey; d Ankara Yıldırım Beyazıt University, Faculty of Dentistry, Department of Oral and Maxillofacial Surgery, Ankara, Turkey

**Keywords:** MRONJ, Osteonecrosis, Platelet rich fibrin, Low level laser therapy, Ultrasonic bone surgery

## Abstract

**Introduction:**

Increased incidence of localized osteonecrosis in the jaw bones caused by bisphosphanate use and reduced quality of life of the patients led researchers to diagnose the disease in the early period and to investigate the effective treatment modality.

**Objective:**

Treatment of medication-related osteonecrosis of the jaw patients is based on individual protocols from clinical experience, as there are no definitive treatment guidelines. In view of the lack of consensus on the effectiveness of medication-related osteonecrosis of the jaw treatments in the literature, the aim of this study was to evaluate the surgical technique described in the treatment of advanced stages of medication-related osteonecrosis of the jaw patients.

**Methods:**

Twenty-one patients affected by Stage 2–3 medication-related osteonecrosis of the jaw were treated with ultrasonic piezoelectric bone surgery for necrotic bone removing, leukocyte and platelet-rich fibrin concentrate obtained from the patient’s peripheral blood and Nd:YAG laser for biostimulation. Success was assessed as the maintenance of full mucosal coverage without signs of residual infection at 1-month (T1), 3-months (T2), 6-months (T3) and 1-year (T4) after surgery. Logistic regressions were used to evaluate the association between the different independent variables and treatment outcomes.

**Results:**

Two Stage 3 patients had delayed healing at 1 month after the operation. Complete mucosal healing was achieved in all patients at the third month. Multivariate analysis demonstrated that different variables were not significantly correlated with delayed healing (*p* > 0.05).

**Conclusion:**

The surgical protocol presented in this study shows promising results for surgical management of advanced stages of medication-related osteonecrosis of the jaw patients.

## Highlights

• Removal of necrotic bone is important in the treatment of medication- related osteonecrosis of the jaw.
• Surgical treatment is more successful than conservative treatment in advanced stages (stage 2–3) of the medication- related osteonecrosis of the jaw.
• Relatively less invasive and supportive treatments are recommended in patients with advanced medication- related osteonecrosis of the jaw.
• Patient follow-up, good oral hygiene and patient motivation increase the success rate of the treatment.

## Introduction

Bisphosphonates (BPs) are used therapeutically to prevent bone complications in multiple myeloma and metastatic bone cancers and also to prevent bone resorption in the treatment of osteoporosis.^1^ BPs act by inhibiting osteoclastic activity, which is generally responsible for bone destruction. It has also been shown that these drugs inhibit osteoblastic activity and diminish the growth and healing of mucosal epithelial cells.^2^ Although BPs provide significant benefits in treatment, Marx reported 30 cases of jawbone osteonecrosis associated with the use of bisphosphonates.^3^

In recent studies, antiresorptive and antiangiogenic drugs (e.g., denosumab, bevacizumab and sunitinib) other than bisphosphonates have been reported to cause bisphosphonates-related osteonecrosis of the jaw (BRONJ)-like lesions. As a result, the American Association of Oral and Maxillofacial Surgeons (AAOMS) recently defined the term “medication-related osteonecrosis of the jaw” (MRONJ) with three criteria. These criteria are presence of antiresorptive or antiangiogenic drug use history, the presence of a clinically exposed bone area longer than 8-weeks, absence of radiotherapy history or history of metastasis localized to the jaw bone.^4^

Increased incidence of localized osteonecrosis in the jaw bones caused by bisphosphanate use and reduced quality of life of the patients led researchers to diagnose the disease in the early period and to investigate an effective treatment modality. In a recent position paper published in 2014, AAOMS has determined the treatment principles for MRONJ according to the stages of the disease.^4^ According to the AAOMS, MRONJ was divided into four different stages. In stage 0, patients had no clinical evidence of necrotic bone, but exhibited nonspecific clinical findings, radiographic changes, and symptoms. In Stage 1, exposed, necrotic bone or fistulas that, when probed, connect to the bone in patients that are asymptomatic and have no evidence of infection. In Stage 2, exposed, necrotic bone or fistulas that connect to the bone, upon probing associated with infection. Infection is evidenced by pain and erythema in the region of exposed bone, with or without purulent drainage and Stage 3, exposed, necrotic bone or fistulas that connect to the bone, upon probing, in patients with pain, infection, and at least one of the following: exposed, necrotic bone extending beyond the region of alveolar bone (i.e., inferior border and ramus in mandible, maxillary sinus, and zygoma in maxilla), which results in pathologic fracture; extraoral fistula; oral antral or oral nasal communication; or osteolysis that extends to the inferior border of the mandible or sinus floor.^4^ Although this stage-specific approach allows a standard treatment protocol in the early stages (Stage 0–1), each patient needs to be evaluated individually in advanced stages (Stage 2–3).^5^ While a non-invasive treatment protocol is recommended in stage 0–1 patients, whose symptoms are milder, there are studies in the literature that surgical treatment is more successful than conservative treatment, especially in advanced stages (Stage 2–3) of the disease.^6^, ^7^, ^8^, ^9^ Severe forms of the disease adversely affect quality of life and produce significant morbidity in patients suffering from these lesions. Although the need for surgical treatment is widely accepted in the advanced stages of MRONJ, there is still some debate about which surgical technique to apply.

In view of the lack of consensus on the effectiveness of MRONJ treatments in the literature, the aim of this study was to evaluate the described surgical procedures to treatment of advanced stages (Stage 2–3) of MRONJ patients.

## Methods

### Patients

In this retrospective cohort study, 21-patients with MRONJ who were referred to our department between 2017 May–2019 May were included. A retrospective analyzed of all followed-up patients diagnosed with MRONJ and treated surgically was performed by examining our department logbooks and databases. The study was approved by the ethics committee of our university (IRB number: 2019/311). All authors read the Helsinki Declaration and followed the guidelines in the study. All patients were informed about treatment options and possible risk of treatment failure. Informed consent was obtained from all participants.

Inclusion criteria were application of the technique defined by the same surgical team patients diagnosed with Stage 2–3 MRONJ according to AAOMS classification, patients who were receiving/received IV BP therapy with zoledronic acid 4 mg per month for at least 3-year for an underlying malignant disease, who followed-up 1-month, 3-months, 6-months and 1-year after surgery. The exclusion criteria were history of head and neck radiotherapy or metastases to the jaw bones, history of previous surgical operations for MRONJ treatment and incomplete follow-up examinations.

### Surgical procedure

**Figure 1:**
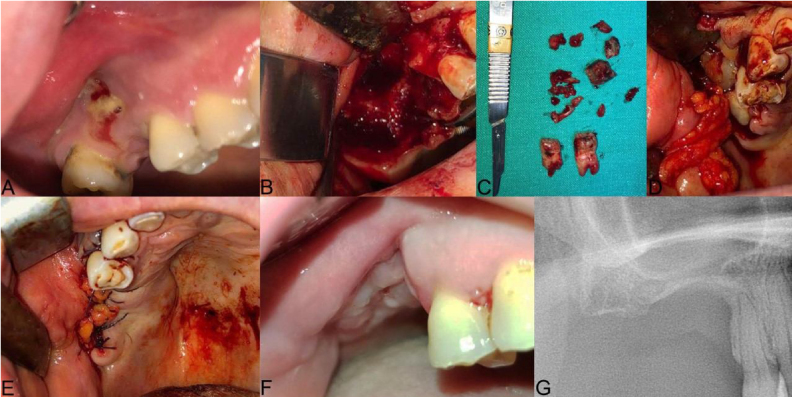
The clinical presentation of medication-related osteonecrosis of the jaw lesion in a 49-year-old female patient with metastatic breast cancer (Stage 3): (A) preoperative picture showing exposed bone surrounded by inflamed swollen mucosal tissue in the right posterior maxilla; (B) oroantral fistula was evident after neurectomy; (C) necrotic bone was completely removed and 15, 17 extracted; (D) the prepared leukocyte- and platelet-rich fibrin is performed under the pedicled buccal fat pad flap to the area of osteonecrosis to contribute to the hard and soft tissue healing; (E) double-layered wound closure was established using pedicled buccal fat pad flap and mucoperiosteal flap; (F and G) post-op 3-months follow-up, clinical healing of the treated lesion without signs of recurrence is evident.

**Figure 2:**
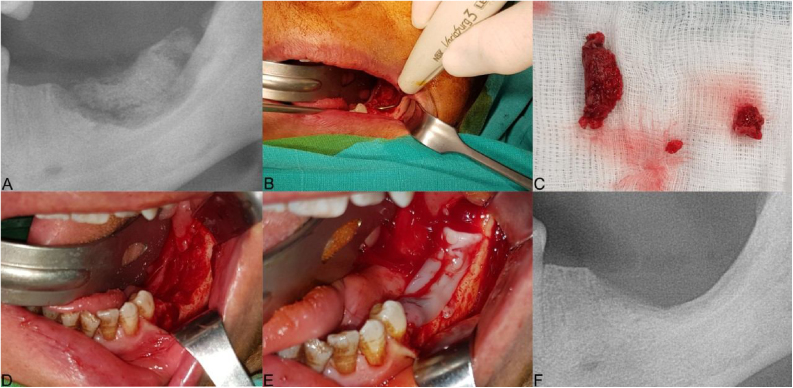
The clinical presentation of medication-related osteonecrosis of the jaw lesion in a 59-year-old female patient with metastatic breast cancer (Stage 2): (A) panoramic image demonstrating bone sequestrum at the left posterior mandibula region; (B) the necrotic bone was removed using ultrasonic piezoelectric bone surgery; (C) necrotic bone was completely removed; (D) debridement boundaries were determined by fresh bleeding from healthy bone; (E) the prepared leukocyte- and platelet-rich fibrin is performed to the area of osteonecrosis to contribute to the hard and soft tissue healing; (F) post-operative panoramic image at 3-months after the operation.

**Figure 3:**
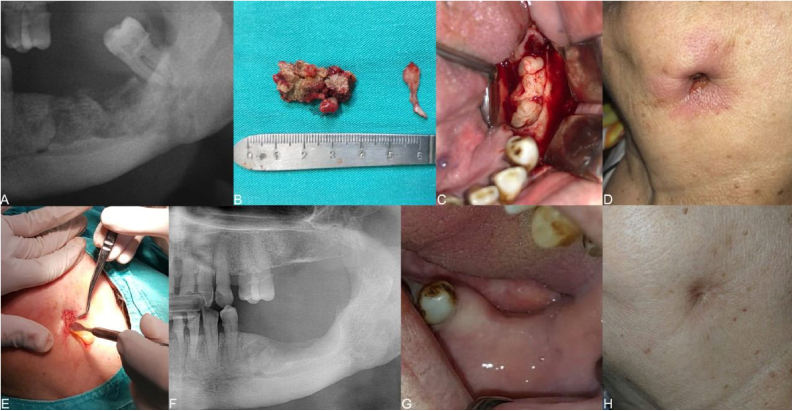
The clinical presentation of medication‐related osteonecrosis of the jaw lesion in a 79-year-old female patient with metastatic breast cancer (stage 3): (A) initial panoramic radiograph of the lesion extending to the mandibular basis in the left posterior mandible; (B) necrotic bone was completely removed; (C) placement of the leukocyte- and platelet-rich fibrin membrane in the surgical area; (D) initial clinical photograph showing a skin sinus tract at the submandibular area; (E) placement of the leukocyte- and platelet-rich fibrin membrane in the skin sinus tract; (F) follow-up at 12-months, postoperative panoramic radiograph of the patient after surgery of the mandibular left molar region; (G) follow-up 3-months, postoperative clinical photograph revealing healing of surgical area; (H) follow-up 3-months, clinical photograph demonstrating healing of the skin sinus tract.

Demographic data, systemic diseases, history of bisphosphanate treatment (type of drug, route of administration, duration of use, underlying disease), smoking habit, diabetes and steroid use were recorded during the first examination. Panoramic radiographs were taken for the first examination of the lesion radiologically. Cone beam computed tomography (CBCT) was used to determine the borders of the lesion more clearly. Clinical and radiological findings based on AAOMS classification, MRONJ stages were defined. After consultation with physicians who prescribed medication, bisphosphonate treatment was discontinued until 2-months after the surgical procedure. Some of the patients had already stopped taking the drug when they came to us for treatment. 1000 mg of amoxicillin/clavulanic acid, 500 mg of metronidazole and 0.12% chlorhexidine digluconate mouthwash were prescribed for 1-week prior to surgery and for 2-weeks postoperative. Surgical procedures were performed under local anesthesia (2 mL of 4% articaine hydrochloride with 1:200,000 epinephrine). Debridement boundaries were determined by fresh bleeding from healthy bone. A pedicled buccal fat pad flap (PBFP) was mobilized in maxillary posterior region surgeries (Fig. 1). In order to expose the necrotic area, crestal incisions were made extending on both sides of the necrosis. In addition to the fistulas, mucosal dehiscence was included in the incision line. The necrotic bone was removed using ultrasonic piezoelectric bone surgery (Fig. 2). The bone surface was smoothed by removing sharp bone edges. After debridement, the leukocyte and platelet-rich fibrin concentrate (L-PRF) obtained from the patient's peripheral blood was centrifuged (IntraSpin™, Intra-Lock System, Boca-Raton, FL, The United States of America) in 10 mL tubes without anticoagulants for 10-minutes at 3000 rpm (Fig. 3). The mucoperiosteal flap was adapted by releasing incisions and closed with 3–0 sutures without tension with apical mattress, locked continuous and simple sutures. The mucosa adjacent to MRONJ lesions was resected before suturing in order to not adversely affect healing. Nd: YAG laser (Fotona-Slovenia) (wavelength 1064 nm, power 1.25 W, frequency 15 Hz, fiere 320 μmin diameter) for biostimulation was applied defocalised at 1–2 mm from the tissue for 1-min and repeated 5 times on post-op 2, 5, 7, 10, 14, 21 and 28th days. Sutures were removed on post-op 14th day. Patients using a prosthesis did not use their prosthesis for 3-months. Then they used their prostheses with soft lining material or new prostheses were made. Patients were examined clinically and radiologically at post op 1, 3, 6 and 12 months. Patients were followed up for at least 12-months and periodic controls are still ongoing.

### Data collection

The results were evaluated in terms of complete mucosal healing. In this study, success was defined by the complete mucosal healing, bone covering and the absence of the symptoms (suppuration, pain, flap dehiscence and hematoma) following surgical treatment. Markers of clinical success were defined as complete mucosal healing without sinusitis, oroantral fistula, extraoral fistula, or exposed bone. The markers of radiological success were accepted as reversing the bone anomaly. Data were collected retrospectively from patient records and surgical documents. All measurements were evaluated at 4 specific time points: 1-month (T1), 3-months (T2), 6-months (T3) and 12-months (T4) after the operation. Healing that occurred four weeks after the operation (T1) was defined as delayed healing.

### Statistical analysis

Statistical evaluations were performed using SPSS 21.0 (Statistical Package for the Social Sciences, Chicago, IL, USA). Results are presented as percentages and continuous variables are presented as mean and standard deviation. Logistic regressions were used to evaluate the association between the different independent variables (age, sex, type of primary disease, stage of MRONJ, size of MRONJ, location of MRONJ, number of BPs applications, chemotherapy, chronic corticosteroids, diabetes mellitus and smoking) and treatment outcomes. Multivariate ordinal logistic regression was performed to determine the different variables that predicted the delayed healing. All data were evaluated at a signiﬁcance level of p < 0.05.

## Results

Of the 21 patients included in the study, 14 were female and 7 were male. The mean age was 68.04 ± 9.82 (range 49–85). Six of the patients were Stage 3 and 15 were Stage 2. Fourteen patients received bisphosphonate treatment for breast carcinoma, 3-patients for prostate carcinoma, 2-patients for lung carcinoma, one patient for kidney cell carcinoma and one patient for multiple myeloma. All patients were using zoledronate with IV route. The mean duration of drug use was 64.76 ± 21.53 months (39–96 months). Six patients had a smoking habit (1-pack per day). Eight of the lesions were in the maxilla and 13 were in the mandible. Factors causing osteonecrosis: 18-patients had tooth extraction, 2-patients had a dental implant and one patient had prosthesis pressure. Some of the patients had already stopped BP medication when they were referred to us. Surgical procedures were performed after 2-months of drug discontinuation in patients receiving medication. The mean duration of drug holiday was 4.52 ± 1.12 months (2–10 months) (supplemental digital content) (Table 1). The response of each patient to treatment was recorded by regular controls. Surgical treatment was successful in all of the patients. In two Stage 3 patients had delayed healing. Complete mucosal healing was achieved in all patients at third months, sixth months and one year (T2–T3–T4). The mean follow-up period was 18.04 ± 2.14 months. A multivariate analysis demonstrated that different variables were not significantly correlated with delayed healing (*p* > 0.05) (supplemental digital content) (Table 2). Note that the CIs are wide, considering the small sample size. The size of the lesions is large and located lingually in two patients with delayed healing. Both patients also have story of corticosteroid use.

**Table 1:** Clinical characteristics of patients included in the study.

Patient number	Sex	Age	Underlying disease	Duration of anti- resorptive drugs	Location of MRONJ	Stages of MRONJ	Drug holiday	Etiologic factors	Outcome	Follow-up
1	F	85	Breast Ca	62 months	33–35	2	4 months	Extraction	Cured	12 months
2	F	62	Bresat Ca	39 months	17,18	3	3 months	Extraction	Cured	19 months
3	F	80	Breast Ca	68 months	33,34	2	2 months	Extraction	Cured	22 months
4	F	79	Breast Ca	84 months	35–37	3	8 months	Extraction	Delayed	25 months
5	M	72	Prostate Ca	51 months	45–47	3	2 months	Extraction	Delayed	14 months
6	F	65	Breast Ca	60 months	35,36	2	7 months	Implant	Cured	18 months
7	F	58	Breast Ca	72 months	44,45	2	5 months	Extraction	Cured	26 months
8	M	49	Breast Ca	66 months	14–17	3	8 months	Extraction	Cured	20 months
9	F	64	Breast Ca	49 months	44,45	2	2 months	Extraction	Cured	28 months
10	M	73	Prostate Ca	64 months	25,26	2	10 months	Extraction	Cured	16 months
11	F	69	Breast Ca	52 months	24,25	2	5 months	Extraction	Cured	13 months
12	M	66	Prostate Ca	96 months	26,27	3	8 months	Extraction	Cured	24 months
13	F	64	Breast Ca	81 months	11–13	2	2 months	Prosthesis	Cured	21 months
14	F	73	Breast Ca	63 months	35–37	2	5 months	Extraction	Cured	15 months
15	M	70	Lung Ca	72 months	46,47	2	3 months	Extraction	Cured	20 months
16	M	70	MM	59 months	32–42	2	2 months	Extraction	Cured	9 months
17	F	68	Breast Ca	48 months	35	2	6 months	Implant	Cured	17 months
18	F	76	Breast Ca	84 months	16,17	3	4 months	Extraction	Cured	12 months
19	F	62	Lung Ca	66 months	46,47	2	2 months	Extraction	Cured	15 months
20	M	65	Kidney Ca	52 months	23	2	4 months	Extraction	Cured	12 months
21	F	59	Breast Ca	72 months	36,37	2	3 months	Extraction	Cured	21 months

MRONJ, medication-related osteonecrosis of the jaw; F, female; M, male; Ca, cancer; MM, multiple myeloma.

**Table 2:** Results of logistic regression analysis examining the effect of different variables on the delayed healing.

Variable	Odds ratio	95% confidence interval	*p*-value
Age	1.429	0.804–3.528	0.726
Sex	2.172	1.273–9.362	0.583
Primary disease	0.971	0.662–15.729	0.224
Stage	3.374	2.451–9.256	0.182
Size	0.718	0.593–2.411	0.164
Location	1.224	0.849–5.712	0.621
Number of BPs applications	1.473	1.318–3.462	0.562
Chemotherapy	3.851	0.092–21.446	0.687
Chronic corticosteroids	2.952	2.128–11.831	0.519
Diabetes *mellitus*	3.125	1.861–19.175	0.337
Smoking habit	1.149	0.857–3.149	0.414

*p* < 0.05 considered significant.

## Discussion

The management of patients affected by MRONJ is based on individual protocols emerging from clinical experience as there are no definitive treatment guidelines. In the concept of stage-specific treatment, consensus has been reached among clinicians trying to find the best treatment for this disease.^5^ Different approaches seem to lead to better results when applied at a particular stage of the disease. According to the results of a recent systematic review, the success rate of surgical treatment in four BRONJ patients varies from 58% to 100%.^10^ Surgical treatment is more appropriate in this patient group since the success rate of conservative treatment is below 50% in advanced stages.^9^, ^11^, ^12^

A non-invasive treatment protocol has emerged in patients with Stage 0–1, where symptoms of AAOMS disease are milder.^4^ However, these patients mostly cannot tolerate the side effects of long-term antibiotic use due to their age, long-term chemotherapy or because of metastatic bone tumors. Recurrence of symptoms after an average of 3-weeks after local or systemic antibacterial and antibiotic therapy led the researchers to alternative treatment methods.^13^ Conservative treatment may provide temporary relief by minimizing symptoms and infections, but osteonecrosis should not be expected to be successfully resolved.^14^ When the exposed necrotic bone is encountered, the effectiveness of conservative treatment cannot be mentioned because the bone tissue is no longer vital and cannot be restored. The aim of this study was therefore to evaluate the surgical technique described in advanced stages of MRONJ patients.

In the literature, discontinuing medication before surgical procedures is a controversial issue.^15^ According to the results of a recent systematic review and meta-analysis, multiple myeloma and osteoporosis patients using alendronate, ibandronate, and zoledronate showed a higher prevalence of complete recovery in the group with drug holiday. Some researchers stated that the drug holiday is unnecessary due to the 11-year half-life of bisphosphonates and the irreversible binding of bone hydroxyapatite crystals and with the interruption of these drugs, pain relapses and bone metastases will increase.^16^ Most of the patients in our study had stopped using the drug when they were referred to us. We did not prefer the drug holiday in other patients.

In MRONJ treatment, complete removal of the necrotic bone with perioperative antibiotic treatment, infection control and smoothing of the sharp bone edges before tension-free wound closure are generally considered as the most appropriate approach for successful recovery.^7^ MRONJ treatment requires surgical debridement until bone appears healthy in terms of structure. In MRONJ lesions, the exposed bone is typically darker and yellowish in color than the unaffected areas. Because of the increased porosity, the necrotic bone is usually softer and is surrounded by sclerotic areas, which in turn are harder and less vascularized. It is thought that bone hemorrhage indicates vital bone that is valid for surgical treatment osteonecrosis and especially MRONJ.^8^, ^17^ Since the removal of necrotic bone is important in the treatment of MRONJ, surgical resections are highly invasive procedures in advanced stage patients, especially Stage 3 patients. Elderly patients with osteoporosis and patients with metastatic malignancies with severe systemic disorders may not tolerate these operations. Therefore, relatively less invasive, and supportive treatments are recommended in patients with advanced MRONJ.^18^ In this study, various treatment methods that have been shown to be effective in the surgical management of MRONJ have been combined and a complete mucosal healing has been achieved in all patients.

To date, several alternatives, and relatively less invasive methods of treatment for MRONJ have also been proposed.^6^ A potential strategy is to use ultrasonic piezoelectric surgery with mouthwashes to remove the necrotic bone. Piezosurgery can remove the necrotic bone with minimal trauma to the soft tissue surrounding the necrotic bone, which may help to eliminate the need for invasive bone resection procedures with saws or rotary instruments.^19^ In addition, piezosurgery allows maintaining the continuity of the vital bone, which may be beneficial for ONJ treatment success, and shows bactericidal effects. Blus et al.^20^ have treated 20 surgical fields of 18-patients with MRONJ using ultrasonic piezoelectric bone surgery combined with antibiotic therapy. Merigo et al.^21^ used ultrasonic bone surgery for sequestrectomy in their combined surgical technique in the treatment of MRONJ patients. We used ultrasonic piezo electric bone surgery to remove the necrotic bone in our patients.

Mucosal wound closure plays an important role in MRONJ treatment. While simple mucoperiosteal closure is sufficient in the early stages, the success of double-layer closure techniques with a pedicled buccal fat pad or myeloid flap has been demonstrated in patients with advanced stage MRONJ patients who show signs of severe bone destruction such as pathological fracture, extraoral fistula, oroantral fistula.^22^ Additional soft tissue layers help for better vascularization and mechanical stability. In addition to the advantages of double-layer protection and high vascularization for nutrition and inflammatory support, PBFP has been revealed to be an abundant source of adipose-derived stem cells. Melville et al.^23^ and Aljohani et al.^24^ reported 23 and 14 Stage 3 MRONJ cases that were effectively treated with PBFP and mucoperiosteal flaps, respectively. In accordance with these studies, we successfully treated MRONJ cases in the maxillary posterior region using PBFP and mucoperiosteal flap in 5-patients. A myeloid flap has been reported with low complication rates and high success rates in wound closure, especially in Stage 3 MRONJ patients.^22^ Especially in lingual lesions, a mucoperisteol flap is insufficient due to the floor of the mouth. In this study, the lesion was located in the lingual mandible in two patients with delayed healing.

Recently, autologous platelet concentrates (APC), platelet rich plasma (PRP), plasma rich in growth factors (PRGF), and L-PRF, have shown promising results in hard and soft tissue regeneration. Application of APCs is used as a supportive therapy in the treatment of MRONJ because it supports angiogenesis and tissue healing by local immunomodulatory properties and platelet factors. According to a recently published systematic review on the effectiveness of platelet concentrates in the prevention and treatment of MRONJ, a success rate of 87.8% was found in cases with APC in addition to surgical treatment and 63.8% in cases with surgery alone. This shows that APCs have promising results in MRONJ treatment.^25^ A new method known as L-PRF was developed in 2006. L-PRF is a physiological agent that provides long-term secretion of growth factors, provides rapid recovery, and reduces the risk of contamination, edema, and postoperative pain. Surgically, it helps homeostasis and prevents flap opening, promoting the remodeling and recovery of both soft and hard tissues. In a single randomized clinical trial examining the effectiveness of PRF in MRONJ treatment, Giudice et al.^26^ treated 47 Stage 2–3 patients in a study in which they evaluated the efficacy of PRF in MRONJ treatment. 24-patients underwent PRF treatment in addition to surgery and 23-patients underwent only surgery without PRF. They compared the groups in terms of mucosal integrity, absence of infection and pain at 1-, 6- and 12-months follow-up and found differences only at first month results. It has been shown that the application of PRF in the surgical treatment of MRONJ may improve the quality of life and reduce pain and postoperative infections limited to short-term follow-up. Based on these studies, we applied L-PRF to the surgical sites as adjuvant therapy in addition the surgical treatment.

Photobiomodulation, known as low dose laser therapy (LLLT), has been used for many years to treat patients with various diseases and conditions. The effects of low-light laser therapy (LLLT) on wound healing, pain relief and nerve regeneration have been reported in vitro and in vivo studies as decreased inflammation and increased collagen and granulation tissue and faster epithelialization.^27^ Laser devices have been described as useful tools for different applications in the treatment of MRONJ, providing biomodulation of both soft and hard tissues as well as the removal of necrotic bone by vaporization.^28^ In a recently published systematic review and meta-analysis, laser-assisted surgery and post-op LLLT applications have been reported to be more successful than traditional surgical methods.^28^ Vescovi et al. reported that Nd:YAG laser can be used for biostimulation in the treatment of BRONJ using a clinical protocol supported by Nd:YAG laser therapy in two separate studies.^29^, ^30^ In our study, we performed Nd:YAG laser for biostimulation in accordance with the protocol of Vescovi et al.^30^

## Conclusions

Because of the pathophysiology of MRONJ is not fully understood and has many risk factors, definitive protocols for treatment have not been established yet. Since the removal of necrotic bone is important in the treatment of MRONJ, surgical resections are highly invasive procedures in advanced stage patients, especially in Stage 3 patients. Therefore, relatively less invasive, and supportive treatments are recommended in patients with advanced MRONJ. In this study, various treatment methods that have shown effectiveness in the surgical management of MRONJ have been combined and a complete mucosal healing has been achieved in all high-risk patients. The treatment is based on surgical procedures, ultrasonic piezoelectric bone surgery for necrotic bone removal, leukocyte and platelet-rich fibrin concentrate (L-PRF) obtained from the patient’s peripheral blood and Nd:YAG laser for biostimulation. The surgical protocol presented in this study shows promising results for surgical management of advanced stages of in high risk MRONJ patients.

## Conflicts of interest

The authors declare no conflicts of interest.
